# Borneol, a messenger agent, improves central nervous system drug delivery through enhancing blood–brain barrier permeability: a preclinical systematic review and meta-analysis

**DOI:** 10.1080/10717544.2018.1486471

**Published:** 2018-10-18

**Authors:** Qun Zheng, Zi-Xian Chen, Meng-Bei Xu, Xiao-Li Zhou, Yue-Yue Huang, Guo-Qing Zheng, Yan Wang

**Affiliations:** a Department of Cardiology, The Second Affiliated Hospital and Yuying Children’s Hospital of Wenzhou Medical University, Wenzhou, PR China;; b Department of Neurology, The Second Affiliated Hospital and Yuying Children’s Hospital of Wenzhou Medical University, Wenzhou, PR China

**Keywords:** Borneol, blood–brain barrier, drug delivery, preclinical evidence, possible mechanisms

## Abstract

To achieve sufficient blood–brain barrier (BBB), penetration is one of the biggest challenges in the development of diagnostic and therapeutic for central nervous system (CNS) disorders. Here, we conducted a systematic review and meta-analysis to assess the preclinical evidence and possible mechanisms of borneol for improving co-administration of CNS drug delivery in animal models. The electronic literature search was conducted in six databases. Fifty-eight studies with 63 comparisons involved 1137 animals were included. Among 47 studies reporting the assessments of CNS drug concentration, 45 studies showed the significant effects of borneol for improving CNS drug delivery (*p*<.05), whereas 2 studies showed no difference (*p*>.05). Nineteen comparisons showed borneol up-regulated BBB permeability (*p*<.05) using brain EB content (*n* = 8), Rh 123 content (*n* = 4), brain imaging agent content (*n* = 2), brain water content (*n* = 1) and observing ultrastructure of BBB (*n* = 4), whereas three studies showed no difference or unclear results. Seven studies reported the safety, in which one study showed borneol was reversible changes in the BBB penetration; six studies showed borneol did not increase co-administration of blood drugs concentration of peripheral tissues (*p* > .05). Effects of borneol are closely associated with inhibition of efflux protein function, releasement of tight junction protein, increasement of vasodilatory neurotransmitters, and inhibition of active transport by ion channels. In conclusion, borneol is a promising candidate for CNS drug delivery, mainly through mediating a multi-targeted BBB permeability.

## 1. Introduction

A key obstacle for therapeutic drugs administered for central nerve system (CNS) disease is passage across the blood–brain barrier (BBB) (Abbott, 2013). The BBB is a specialized non-permeable barrier constituted by endothelial cells, a basal lamina and astrocytic endfeet (Zlokovic, 2008). It serves a predominant role in regulating supply of essential nutrients to the brain as well as protecting the CNS from many potentially harmful compounds (Abbott et al., 2010). The property of selective impermeable BBB is mainly due to the presence of tight junctions between adjacent endothelial cells and the existence of various BBB transporters, e.g. efflux transporters P-glycoprotein (P-gp). The tight junctions are against the access of about 100% of large-molecule neurotherapeutics and ∼98% of all small-molecule drugs to the brain (Pardridge, 2005). The BBB transporters are against the accumulation of a wide range of drugs in brain (Demeule et al., 2002). Thus, the BBB maintains the brain homeostasis and also inhibits the entry of potentially useful diagnostic and therapeutic agents, which consequently restricts the therapeutic effects of majority of drugs on many CNS disorders (Abbott et al., 2006).

The past 30 years have seen a great deal of research on the CNS drug delivery, and several strategies have been tried to deal with the problem (Banks, 2016). For example, highly invasive strategies, i.e. intracerebral or intracerebroventricular administration are useful for local CNS delivery in specific cases e.g. in well-defined tumors, but they are risky, costly, and of limited value for the administration of therapeutic agents that are directed toward less localized diseases such as diffused tumors, Alzheimer’s disease, and multiple sclerosis (Garcia et al., 2005). Furthermore, higher concentrations of drug facilitate entry, but efficacy is limited by dose-dependent toxicity of peripheral tissues (Banks, 2016). What is more, approaches that disrupt an intact BBB in an attempt to let in a candidate drug also let in circulating substances that are normally excluded by the BBB and can be quite toxic to the CNS (Kroll & Neuwelt, 1998). Thus, numerous intravascular drugs delivery strategies which consider BBB as a therapeutic target have been proposed gradually and tested in hope of enhancing BBB penetration instead of disrupting BBB to achieve a widespread transport of the infused drug across the whole brain parenchyma (Tosi et al., 2008). Up to now, a number of intravascular strategies have been explored to improve the transport of drug across BBB, such as osmotic and chemical modifications of BBB, enhanced transcellular transport, nanoparticle carriers, and cell-based drug delivery (Hersh et al., 2016). This is a promising but difficult area of drug development, as specific features, advantages, and limitations in every strategy (Hersh et al., 2016), and few drugs have been successfully applied to the clinic (Zhang et al., 2017). This complexity confounds simple strategies for drug delivery to the CNS, but provides a wealth of opportunities and approaches for drug development (Banks, 2016).

Borneol, highly lipid-soluble bicyclic terpene chemicals extracted from *Cinnamomum camphora* (L.) Presl. and *Blumea balsamifera* (L.) DC. or chemically transformed on the basis of camphor and turpentine oil (State Pharmacopoeia Committee, 2010), is widely used as a messenger drug in many traditional Chinese herbal prescriptions such as Angong Niuhuang pill, a well-known formula for treating stroke (Guo et al., 2014). According to traditional Chinese medicine (TCM) Emperor-Minister-Assistant-Courier theory, this principle guides the combination of multiple herbal medicines in a specific manner when creating TCM compound prescriptions. Borneol is classified as a ‘Courier herb’ that guides the herbs upward to target organ, especially in the upper part of the body, such as the brain. This studies showed that borneol is not only an effective penetration enhancer through corneal (Yang et al., 2009), intestinal mucosa (Zhang et al., 2012), and nasal cavity mucosa (Lu et al., 2011) but also an effective BBB penetration enhancer for a greater access of drug to the brain (Wang et al., 2014). The increased CNS concentrations of carbamazepine and valproate after the co-administration of borneol in epileptic patients with few side effects have been reported in clinical trials (Xu et al., 2016; Armulik et al., 2010). However, insufficient evidence and unknown mechanism limited the application of borneol in clinic (Zhang et al., 2017). Thus, we conducted a preclinical systematic review to provide the preclinical evidence and possible mechanisms of borneol on up-regulation of BBB permeability to enhance CNS drug concentrations.

## 2. Methods

### 2.1. Search strategy

The systematically electronic literature search was conducted *via* PubMed, Chinese National Knowledge Infrastructure, VIP Database, Wanfang database, and Chinese Biomedical Database from their inceptions to December 2017. The search terms were as follows: ‘borneol OR camphol’ AND ‘blood brain barrier’ in Chinese or in English. All searches were limited to animal studies.

### 2.2. Eligibility criteria

Studies of borneol for CNS drug delivery through enhancing BBB permeability *in vivo* were included. There was no restriction on animal species or publication status. Eligibility criteria were: (Abbott, 2013) borneol for animal, regardless of its mode, dosage and the administration frequency; (Zlokovic, 2008) the primary outcome measures were the co-administration of drug concentrations in CNS, and the second outcome measures were the safety of borneol, the various indexes of BBB permeability, and possible mechanisms of borneol for enhancing BBB permeability; (Abbott et al., 2010) interventions for control group were isasteric and nonfunctional liquid (normal saline) or no treatment. Exclusion criteria were predefined as follows: (Abbott, 2013) case reports, reviews, abstracts, news, comments, editorials, and *in vitro* studies; (Zlokovic, 2008) compared with medicine or another agent with potential similar effect; (Abbott et al., 2010) was not tested on the primary and/or second outcome measures; (Pardridge, 2005) lack of control group; (Demeule et al., 2002) duplicate publication.

### 2.3. Data extraction

Two authors independently reviewed each included study and extracted following aspects of details: (Abbott, 2013) name of first author, year of publication and method of anesthesia and/or model; (Zlokovic, 2008) details (species, number, sex, and weight) of animals for each study; (Abbott et al., 2010) the use of anesthesia in the experiment and the methods to establish animal models; (Pardridge, 2005) the information on the method of administration was obtained from both treatment and control group including drug, dose, mode and frequency; (Demeule et al., 2002) the outcome measures and samples for individual comparison were included. A comparison was defined as the qualitative and/or quantitative assessments of co-administration of drug concentrations in CNS and/or the safety of borneol and/or the various indexes of BBB permeability in treatment and corresponding control group after the administration of borneol or vehicle with a given dose, mode, and frequency. In case of lack of vehicle group, the group receiving no adjunct intervention was used as control group for individual comparison. If a drug concentration was used for outcome assessment, both the drug and the method of drug administration were obtained. All available data from quantitative assessments of primary and second outcomes were extracted for every comparison including mean outcome and standard deviation (Abbott et al., 2006). The efficacy result was summarized as increased or decreased according to whether a significantly increasing or decreasing outcomes in each study. If there was no statistical difference of effects of borneol between treatment and control groups, the efficacy results were summarized as no difference. In instances of absence of statistical analysis within comparison as well as available original data, the efficacy result of the comparison was listed as “increased?” or “decreased?”

### 2.4. Quality of evidence

Two authors independently conducted the quality assessment of included studies according to a ten-item modified scale with minor modification: (Abbott, 2013) peer-reviewed publication; (Zlokovic, 2008) statement of physiological parameters control, such as temperature; (Abbott et al., 2010) random allocation; (Pardridge, 2005) blinded conduct of the experiments; (Demeule et al., 2002) blinded assessment of outcome; (Abbott et al., 2006) use of anesthetic without significant intrinsic neuroprotective activity; (Banks, 2016) appropriate animal and/or model (brain tumor model, epilepsy, intracranial infection, cognitive dysfunction or Parkinsonism); (Garcia et al., 2005) sample size calculation; (Kroll & Neuwelt, 1998) compliance with animal welfare regulations; (Tosi et al., 2008) statement of potential conflict of interests (Landis et al., 2012; Macleod et al., 2004).

### 2.5. Statistical analysis

The statistical analysis was conducted *via* RevMan version 5.3 software in Copenhagen, Denmark. To estimate the effect of borneol on CNS drug delivery and/or BBB permeability across studies, a summary statistic was calculated for each comparison with 95% confidence intervals by using the random effects method. When the outcome measurements in all included studies in meta-analysis were based on the same scale, weighted mean difference (WMD) was calculated as a summary statistic. On the contrary, when the same outcome measurements were measured in a variety of ways across studies in meta-analysis, standardized mean differences (SMD) was used as a summary statistic. Heterogeneity between study results was investigated based on a standard chi-square test and *I*^2^ statistic. A probability value .05 was considered statistically significant.

## 3. Results

### 3.1. Study selection

**Figure 1.**
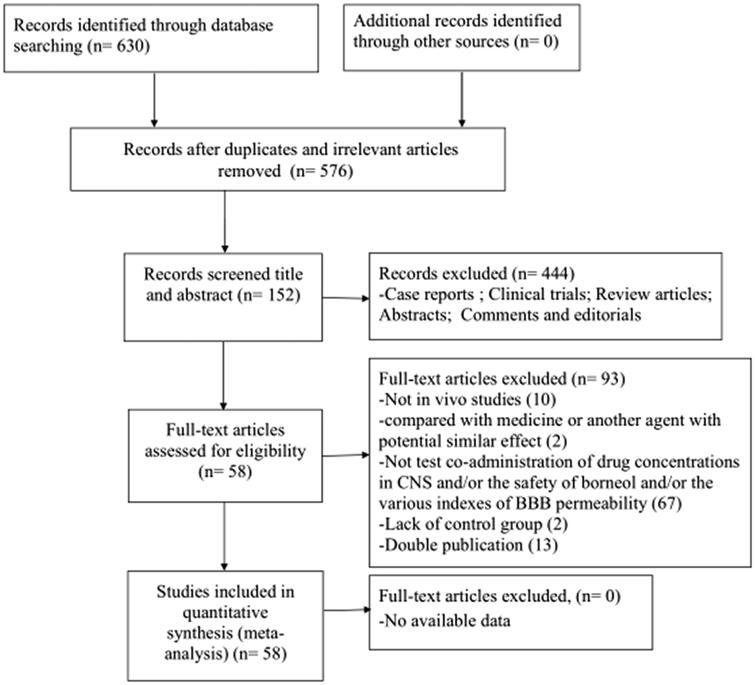
Summary of the process for identifying candidate studies.

A total of 630 potentially relevant articles were identified, of which 54 were reduplicated and irrelevant articles. By reviewing titles and abstracts, 444 studies were excluded for at least one of following reasons: (Abbott, 2013) case reports, reviews, abstracts, news, comments, and editorials; (Zlokovic, 2008) not test the effect of borneol on BBB permeability; (Abbott et al., 2010) not *in vivo* studies. After examining the remaining 152 studies through reading the full text, we removed 93 records. Of which, 18 studies were lack of outcome assessments for BBB integrity, 67 studies did not test on co-administration of drug concentrations in CNS and/or the safety of borneol and/or the various indexes of BBB permeability, 2 studies compared with medicine or another agent with potential similar effect, 2 studies were lack of control group, 10 studies were *in vitro* studies, and 13 studies were duplicate publications. Ultimately, 58 studies (Wang et al., 1992; Liang et al., 1993; Liu et al., 1994; Xu & Wang, 1995; Dong et al., 2002; Lin et al., 2003; Jia et al., 2004; Wu et al., 2004; Zhang et al., 2005; Chen, 2005; Zhou et al., 2005; Wang, 2006; Wang et al., 2006; Zheng et al., 2007; Xiao et al., 2007; Chen et al., 2007; Zhang et al., 2007; Liu & Gao, 2007; Lin et al., 2008; Liu et al., 2016; Zhou et al., 2008; Shi & Zhao, 2008; Li et al., 2008; Liu et al., 2008; Ge et al., 2008; Gao et al., 2009; Wu et al., 2009; Wang et al., 2009; Xiao & Ping, 2009; Chai et al., 2009; Zhu, 2009; Wei et al., 2010; Zhang, 2011; Wu, 2011; Zhang et al., 2011; Wang et al., 2011; Yu et al., 2011; Wu et al., 2011; Dong et al., 2012; Yu et al., 2012; Wang et al., 2012; Cao, 2013; Yu et al., 2013; Diao et al., 2013; Huang et al., 2013; Zhang, 2014; Xin et al., 2014; Liu, 2015; Zhang et al., 2015; Guo et al., 2015; Yu et al., 2015; Zhao et al., 2015; Ren, 2016; Wei, 2016; Wu, 2016; Tang et al., 2016; Hou et al., 2016; Yin et al., 2017) were selected for eligibility (Figure 1).

### 3.2. Study characteristics

Fifty-eight studies reported effect of borneol CNS drug delivery and/or the BBB permeability involved 1137 animals. Eleven species were used, including Sprague-Dawley (SD) rats (*n* = 316) (Xu & Wang, 1995; Lin et al., 2003; Liu et al., 2008; Gao et al., 2009; Chai et al., 2009; Zhu, 2009; Zhang, 2011; Zhang et al., 2011; Dong et al., 2012; Yu et al., 2012; Yu et al., 2013; Diao et al., 2013; Zhang et al., 2015; Guo et al., 2015; Hou et al., 2016), Wistar rats (*n* = 198) (Liang et al., 1993; Liu et al., 1994; Dong et al., 2002; Jia et al., 2004; Wang et al., 2006; Xiao et al., 2007; Gao et al., 2009; Zhang et al., 2011; Xin et al., 2014; Zhao et al., 2015; Ren, 2016), Kunming mice (*n* = 298) (Xu & Wang, 1995; Dong et al., 2002; Jia et al., 2004; Li et al., 2008; Wu et al., 2009; Wu, 2011; Yu et al., 2011; Wang et al., 2012; Huang et al., 2013; Wei, 2016; Tang et al., 2016), ICR mice (*n* = 58) (Chen, 2005; Wang et al., 2006; Zhou et al., 2008), Balb/c mice (*n* = 6) (Zhang, 2011), NIH rats (*n* = 20) (Yu et al., 2015), FVB rats (*n* = 12) (Wu, 2016), C57BL/6 mice (*n* = 20) (Yin et al., 2017), New Zealand rabbits (*n* = 44) (Wang et al., 1992; Liang et al., 1993; Zheng et al., 2007; Liu, 2015), Japanese White Rabbits (*n* = 92) (Zhou et al., 2005; Zhang et al., 2007; Liu & Gao, 2007; Shi & Zhao, 2008; Li et al., 2008; Gao et al., 2009), Guinea pigs (*n* = 20) (Zhang et al., 2005) and the remaining animals (*n* = 58) (Wu et al., 2004; Chen et al., 2007; Liu et al., 2008; Wang et al., 2009; Xiao & Ping, 2009) that reported as mouse or rabbit but without species details. The weight of rats ranged from 150 to 350 g, the weight of mice ranged from 15 to 30 g and the weight of rabbits ranged from1.8g to 3.0 kg. Chloral hydrate was used in 19 studies (Chen et al., 2007; Liu & Gao, 2007; Li et al., 2008; Gao et al., 2009; Zhu, 2009; Zhang, 2011; Dong et al., 2012; Yu et al., 2012; Cao, 2013; Huang et al., 2013; Xin et al., 2014; Guo et al., 2015; Hou et al., 2016), pentobarbital in 5 studies (Wang et al., 1992; Zhang et al., 2005; Wang, 2006; Wang et al., 2009; Chai et al., 2009), urethane in 3 studies (Zhou et al., 2005; Zheng et al., 2007; Shi & Zhao, 2008), ether in 1 study (Wei, 2016), avertin in 1 study (Wu, 2016), while no information on anesthetics in the rest 29 studies. As for the method of administration, 21 studies (Wang et al., 1992; Liu et al., 1994; Dong et al., 2002; Lin et al., 2003; Wu et al., 2004; Zhang et al., 2005; Zhou et al., 2005; Wang et al., 2006; Zhou et al., 2008; Ge et al., 2008; Zhu, 2009; Wu, 2011; Yu et al., 2011; Dong et al., 2012; Cao, 2013; Huang et al., 2013; Xin et al., 2014; Zhang et al., 2015) used synthetic borneol, 3 studies (Chen, 2005; Yu et al., 2013; Yin et al., 2017) used L-borneol, 11 studies declared the administration of natural borneol (Chen et al., 2007; Liu & Gao, 2007; Shi & Zhao, 2008; Liu et al., 2008; Gao et al., 2009; Guo et al., 2015; Ren, 2016; Tang et al., 2016) but without reporting the type of borneol, and the remaining studies used borneol without further information provided. Eighteen studies conducted more than two dose gradients of borneol. Among them, 10 studies (Lin et al., 2003; Chen, 2005; Ge et al., 2008; Wang et al., 2011; Yu et al., 2013; Zhang et al., 2015; Guo et al., 2015; Ren, 2016; Wei, 2016) investigated two dose groups, 7 studies (Zhang et al., 2005; Wang et al., 2006; Liu et al., 2016; Wang et al., 2011; Liu, 2015; Tang et al., 2016; Yin et al., 2017) investigated three dose groups, 2 studies (Dong et al., 2002; Zhu, 2009) investigated four dose groups and 1 study (Yin et al., 2017) investigated five dose groups. The mode of borneol application involved oral gavage in 48 studies, intravenous injection in 4 studies (Wang et al., 2006; Wu et al., 2009; Zhang et al., 2015; Hou et al., 2016), nasal administration in 4 studies (Zhang et al., 2005; Liu et al., 2008; Chai et al., 2009; Liu, 2015) and acupoint injection in 1 study (Lin et al., 2003). The frequency of borneol treatment varied from once only (Wang et al., 1992; Xu & Wang, 1995; Lin et al., 2003; Liu et al., 2016; Shi & Zhao, 2008; Chai et al., 2009; Zhang, 2011; Wu, 2011; Wu et al., 2011; Yu et al., 2012; Cao, 2013; Diao et al., 2013; Liu, 2015; Guo et al., 2015; Wei, 2016; Hou et al., 2016) to once daily for the duration of 3–14 d (Yu et al., 2011; Wang et al., 2012; Yu et al., 2015; Zhao et al., 2015). Borneol compared with vehicle in 33 studies and with no adjunct intervention in other 25 studies (Lin et al., 2003; Chen, 2005; Chen et al., 2007; Lin et al., 2008; Liu et al., 2008; Wu et al., 2009; Chai et al., 2009; Wei et al., 2010; Zhang, 2011; Yu et al., 2011; Wang et al., 2012; Xin et al., 2014; Zhang et al., 2015; Yu et al., 2015; Ren, 2016; Wu, 2016; Hou et al., 2016; Yin et al., 2017). About outcomes for assessing CNS drug delivery and/or BBB permeability, 47 studies used the CNS drug concentration to assess the effects of borneol for CNS drug delivery, including 17 studies (Wang et al., 2006; Xiao et al., 2007; Zhu, 2009; Zhang, 2011; Zhang et al., 2011; Wang et al., 2011; Wu et al., 2011; Yu et al., 2012; Zhang, 2014; Zhang et al., 2015; Guo et al., 2015; Zhao et al., 2015; Wei, 2016; Tang et al., 2016; Yin et al., 2017) reporting the brain concentration, 25 studies (Liu et al., 1994; Dong et al., 2002; Jia et al., 2004; Wu et al., 2004; Chen, 2005; Wang, 2006; Lin et al., 2008; Zhou et al., 2008; Liu et al., 2008; Wu et al., 2009; Chai et al., 2009; Zhang, 2011; Wu et al., 2011; Cao, 2013; Xin et al., 2014; Guo et al., 2015; Wei, 2016) reporting the brain to serum concentration ratio, 7 studies (Chen et al., 2007; Liu & Gao, 2007; Gao et al., 2009; Wei et al., 2010; Diao et al., 2013; Liu, 2015) reporting the cerebrospinal fluid (CSF) concentration, 4 studies (Zhou et al., 2005; Zhang et al., 2007; Shi & Zhao, 2008; Li et al., 2008) reporting the CSF to serum concentration ratio of the drug, and 6 studies (Wu et al., 2009; Yu et al., 2012; Wang et al., 2012; Cao, 2013Diao et al., 2013; Xin et al., 2014) reporting the blood drug concentration. In addition, nine studies (Liang et al., 1993; Xu & Wang, 1995; Lin et al., 2003; Zhang et al., 2005; Zhu, 2009; Yu et al., 2011; Wu et al., 2011; Huang et al., 2013; Yin et al., 2017) performed the quantitative assessments of brain for EB, and four studies (Yu et al., 2011; Wang et al., 2012; Yu et al., 2013; Wu, 2016) for rhodamine 123 (Rh 123), four studies (Zhang et al., 2007; Ge et al., 2008; Yu et al., 2011; Yu et al., 2013) reported the ultrastructure of BBB, two studies used imaging such as CT (Wang et al., 1992) and immunofluorescence image (Zhang, 2011), and one study (Wang et al., 2011) for water content (Table 1). About possible mechanisms of borneol for enhancing BBB permeability, 7 studies (Xiao et al., 2007; Chen et al., 2007; Zhang et al., 2011; Wang et al., 2012; Cao, 2013; Diao et al., 2013; Yin et al., 2017) refer to 5-hydroxytryptamine and histamine, 10 studies (Xiao et al., 2007; Chen et al., 2007; Zhu, 2009; Yu et al., 2011; Wang et al., 2012; Diao et al., 2013; Yu et al., 2015; Ren, 2016; Tang et al., 2016; Yin et al., 2017) refer to P-gp, 6 studies (Chen, 2005; Xiao et al., 2007; Chen et al., 2007; Zhou et al., 2008; Yu et al., 2011; Diao et al., 2013) refer to NOS, 3 studies (Wang et al., 2009; Chai et al., 2009; Yu et al., 2011) refer to tight junction, 1 study (Wu, 2016) refer to a chloride-permeable channel CIC-3, and 1 study (Yu et al., 2013) refer to multidrug resistance 1a (Mdr1a), multidrug resistance 1 b (Mdr1b) and multidrug resistance protein 1 (Mrp1).

**Table 1.** Summary the efficacy of borneol for improving central nervous system drug delivery.

Study	Species (sex, *n* = experimental / control group)	Weight	Anesthetic + methods to establish animal models	Method of administration (drug, dose, mode, frequency)		Outcome measures and samples	Efficacy result	
(author, years)				Treatment group	Control group			
Wang et al., 1992	New Zealand rabbits, NS (NS/NS)	2.0–2.5 kg	2% pentobarbital sodium (30 mg/kg, iv)	Synthetic borneol, 1.5 g/kg, ig, once	Same volume of normal saline, ig, once	CT	Increased	
Liang et al., 1993a	New Zealand rabbits, male and female (6/6)	1.8–2.4 kg	NS	Synthetic borneol, 1.5 g/kg, ig, once	Same volume of normal saline, ig, once	The brain concentration of EB	Increased?	
Liang et al., 1993b	Wistar rats, male and female (20/20)	150–180 g	NS	Synthetic borneol, 1 g/kg, ig, once	Same volume of normal saline, ig, once	The brain concentration of EB	Increased?	
Liu et al., 1994	Wistar rats, male and female (14/12)	180–200 g	NS	Synthetic borneol, 1 g/kg, ig, once before administration of the drug	Same volume of normal saline, ig, once before administration of the drug	The brain concentration of Gentamycin (Gentamycin, 3.5 mg/kg, CVI)	Increased	
Xu & Wang, 1995a	SD rats, NS (8/8)	250–350 g	NS	Borneol, 1.5 g/kg, ig, once before administration of the drug	Same volume of liquid paraffin, ig, once before administration of the drug	The brain to serum concentration ratio of Sul (Sul, 200 mg/kg, iv)	Increased	
Xu & Wang, 1995b	Kunming mice, NS (10/10)	22–26 g	NS	Borneol, 1.5 g/kg, ig, once before administration of the drug	Same volume of liquid paraffin, ig, once before administration of the drug	The brain concentration of EB	ND	
Xu & Wang, 1995c	Kunming mice, NS (10/10)	22–26 g	NS	Borneol, 0.5 g/kg, ig, once daily for 4 d before administration of the drug	Same volume of liquid paraffin, ig, once daily for 4 d before administration of the drug	The brain concentration of EB	Increased	
Dong et al., 2002a	Wistar rats, male (6/6)	200 ± 5.25 g	NS	Synthetic borneol, 0.3 g/kg, ig, once daily for 4 d before administration of the drug	Same volume of liquid paraffin, ig, once daily for 4 d before administration of the drug	The brain concentration of Pt^2+^ (Cisplatin, 7 mg/kg, ip)	Increased	
Dong et al., 2002b	Kunming mice, male (6/6)	19.0 ± 0.54 g	NS	Synthetic borneol, 0.125, 0.25, 0.50, 1.00 g/kg, ig, once daily for 4 d before administration of the drug	Same volume of liquid paraffin, ig, once daily for 4 d before administration of the drug	The brain concentration of Pt^2+^ (Cisplatin, 15 mg/kg, ip)	Increased	
Lin et al., 2003	SD rats, male and female (9/10)	280 ± 32 g	NS	Synthetic borneol, 0.4, 0.8 g/ kg, AI at GV 15, once	No adjunctive intervention	The brain concentration of EB	Increased	
Jia et al., 2004	Wistar rats, NS, (10/10)	200 ± 20 g	NS	Borneol, NS, ig, once at 1 h before administration of the drug	Same volume of liquid paraffin, ig, once at 1 h before administration of the drug	The brain concentration of Cisplatin (Cisplatin, 1 mg/kg, ip)	Increased	
Wu et al., 2004	Mice, male (5/5)	20–25 g	NS	Synthetic borneol, 0.6 g/kg, ig, once at 15 min before administration of the drug	Same volume of 1% CMC-Na, ig, once at 15 min before administration of the drug	The brain concentration of Rif (Rif, 182 mg/kg, ig )	Increased	
Zhang et al., 2005	Guinea pigs, male and female (10/10)	200 ± 20 g	3% pentobarbital sodium (NS, ip)	Synthetic borneol 0.0005, 0.001, 0.002 g per animal, in, once	Same volume of liquid paraffin, in, once	The brain concentration of EB	Increased	
Chen, 2005	ICR mice, male and female (5/5)	25 ± 2 g	NS	L-Borneol, 0.0003, 0.0006 g/kg, ig, once	No adjunctive intervention	The brain concentration of paeonol (paeonol, 100 mg/kg, ig)	ND	
Zhou et al., 2005	Japanese white rabbits, male and female (5/5)	2.6 ± 0.22 kg	Urethane (NS, NS)	Synthetic borneol,0.75 g/kg, ig, once before administration of the drug	Same volume of 20% CMC-Na, ig, once before administration of the drug	The CSF to serum concentration ratio of CBZ, ECBZ (CBZ, 40 mg/kg, ig)	Increased	
Wang, 2006	ICR mice, male and female (6/6)	22 ± 2 g	NS	Synthetic borneol, 0.15, 0.3, 0.6 g/kg, ig, once before administration of the drug	Same volume of PEG 400, ig, once before administration of the drug	1. The brain concentration of Clindamycin (Clindamycin, 40 mg/kg, CVI)	Increased	
						2. The brain concentration of amantadine hydrochloride (amantadine hydrochloride, 10 mg/kg, CVI)	Increased	
						3. The brain concentration of fentanyl citrate (fentanyl citrate, 1 mg/kg, CVI)	Increased	
Wang et al., 2006	Wister rats, NS (6/6)	300 ± 50 g	Pentobarbital sodium (NS, NS)	Borneol, 1 g/kg, iv, once	Same volume of 95% ethanol, iv, once	The brain concentration of TMP (TMP, 10 mg/kg, iv)	Increased	
Zheng et al., 2007	New Zealand rabbits, male and female (6/6)	2.0–2.2 kg	Urethane (NS, NS)	Borneol, 0.18 g/kg, ig, once	Same volume of 2% CMC-Na, ig, once	The CSF to serum concentration ratio of Danshensu (*Salvia miltiorrhiza*, 10 g/kg, ig)	Increased	
Xiao et al., 2007	Wistar rats, male (8/8)	250 ± 20 g	NS	Borneol, 1.5 g/kg, ig, once at 1 h before administration of the drug	Same volume of liquid paraffin, ig, once at 1 h before administration of the drug	The brain concentration of As2O3 (As2O3, 0.9 mg/kg, ia)	Increased	
Chen et al., 2007	Rabbits, male and female (6/6)	2.5 ± 0.2 kg	20% choral hydrate (800 mg/kg, ip)	Natural Borneol, 0.7 g/kg, ig, once after administration of the drug	No adjunctive intervention	The CSF concentration of SV (SV, 40 mg/kg, ig and 14 mg/kg, ivgtt)	Increased	
Zhang et al., 2007	Japanese white rabbits, male and female (10/10)	2.5 ± 0.4 kg	20% choral hydrate (1.2 g/kg, ip)	Borneol, 0.7 g/kg, ig, once after administration of the drug	Same volume of 75% ethanol, ig, once after administration of the drug	1. The CSF concentration of Vs (Vs, 40 mg/kg, iv and ivgtt)	Increased	
						2. The ultrastructure of BBB		
Liu & Gao, 2007	Japanese white rabbits, male and female (NS/NS)	2.5 ± 0.4 kg	20% choral hydrate (NS, NS)	Natural Borneol, 0.7 g/kg, ig, once after administration of the drug	Same volume of 75% ethanol, ig, once after administration of the drug	The CSF concentration of SV (SV, 40 mg/kg, iv)	Increased	
Lin et al., 2008	Kunming mice, NS (5/5)	25 ± 5 g	NS	Borneol, 0.002, 0.010, 0.050 g/kg, ig, once	No adjunctive intervention	The brain concentration of SF (SF, 200 mg/kg, ig)	Increased	
Liu et al., 2008	SD rats, male and female (5/5)	190–210 g	NS	Borneol, 0.009 g/kg, ie, once	No adjunctive intervention	The brain concentration of ligustrazine (ligustrazine, 50 mg/kg, ie)	Increased	
Zhou et al., 2008	ICR mice, male and female (6/6)	NS	NS	Synthetic borneol, 0.75 g/kg, ig, once daily for 5 d before administration of the drug	Same volume of corn embryo oil, ig, once daily for 5 d before administration of the drug	The brain concentration of CBZ, ECBZ (CBZ, 760 mg/kg, ig)	Increased	
Shi & Zhao, 2008	Japanese white rabbits, male and female (8/8)	3.0 ± 0.5 kg	20 % urethane (1.0–1.6 g/kg, iv)	Natural Borneol, 0.7 g/kg, ig, once at 1 h before administration of the drug	Same volume of 75% ethanol, ig, once at 1 h before administration of the drug	The CSF to serum concentration ratio of ACNU (ACNU, 4 mg/kg, iv)	Increased	
Li et al., 2008	Japanese white rabbits, Male and female (16/14)	2.5 ± 0.5 kg	20% choral hydrate (1.0–1.2 g/kg, ip)	Natural Borneol, 0.7 g/kg, ig, once at 1 h before administration of the drug	Same volume of 75% ethanol, ig, once at 1 h before administration of the drug	The CSF to serum concentration ratio of TMZ (TMZ, 12 mg/kg, ig)	Increased	
Liu et al., 2008	Rabbits, male and female (6/6)	1.8–2.5 kg	NS	Natural Borneol, 0.18 g/kg, ig, once	Same volume of 2% CMC-Na, ig, once after administration of the drug	The brain concentration of Danshensu (*Salvia miltiorrhiza*, 10 g/kg, ig)	Increased	
Ge et al., 2008	SD rats, male and female (5/5)	260–310 g	NS	Synthetic borneol, 1.5, 1.95 g/kg, ig, once	Same volume of liquid paraffin, ig, once	The ultrastructure of BBB	Increased	
Gao et al., 2009	Japanese white rabbits, male and female (6/6)	2.5 ± 0.5 kg	20% choral hydrate (700–800 mg/kg, ip)	Natural Borneol, 0.7 g/kg, ig, once at 1 h before administration of the drug	Same volume of 75% ethanol, ig, once at 1 h before administration of the drug	The CSF concentration of methotrexate (methotrexate, 100 mg/kg, iv)	Increased	
Wu et al., 2009	Kunming mice, male and female (3/3)	20 ± 2 g	NS	Borneol, NS, iv, once	No adjunctive intervention	1. The brain concentration of AZT (AZTP-CL, 30 mg/kg, NS)	Increased	
						2. The blood concentration of AZT (AZTP-CL, 30 mg/kg, NS)	ND	
Wang et al., 2009	Rabbits, male and female (6/6)	1.8–2.2 kg	Phenobarbitone (45 mg/kg, ip)	Borneol, 0.085 g/kg, ig, once	No adjunctive intervention	The brain concentration of Notoginsenoside R1, ginsenoside Rg1 and Re (Panax notoginseng 15.0 g/kg, ig)	Increased	
Xiao & Ping, 2009	Mice, NS (6/6)	NS	NS	Borneol, 0.03 g/kg, ig, once	No adjunctive intervention	The brain concentration of TMPP (TMPP, 37.5 mg/kg, ig)	Increased	
Chai et al., 2009	SD rats, male (5/5)	320 ± 20 g	Pentobarbital sodium (45 mg/kg, ip)	Borneol, 0.00011 g/kg, ie, once	No adjunctive intervention	The brain concentration of NT-NP (NT-NP, 60 µg/kg, ie)	Increased	
Zhu, 2009	SD rats, male and female (10/10)	250–300 g	10% chloral hydrate (350 mg/kg, CVI)	Synthetic borneol, 0.125, 0.25, 0.50, 1.00 g/kg, ig, once daily for 4 d	Same volume of liquid paraffin, ig, once daily for 4 d	1. The brain concentration of EB	Increased	
						2. The concentration of drug (VCR, 1 mg/kg,CVI)	Increased	
Wei et al., 2010	Wistar rats, male (4/4)	260–300 g	10% choral hydrate (3.45 g/kg, ip)	Borneol, 27 g/kg, ig, once daily for 7 d	No adjunctive intervention	The CSF concentration of ceftriaxone (ceftriaxone, 180 g/kg, im, once daily for 7 d)	Increased	
Zhang et al., 2011 (1)a	Balb/c mice, male (3/3)	18–22 g	10% chloral hydrate (0.4 g/kg, ip)	Borneol, 0.006 g per animal, ig, once after administration of the drug	No adjunctive intervention	Immunofluorescence image	Increased	
Zhang et al., 2011 (1)b	SD rats, male (3/3)	200 ± 10 g	NS	Borneol, 0.001 g/kg, ig, once after administration of the drug	No adjunctive intervention	The brain concentration of Hup (NP-Hup A or Apr-NP-Hup A,, 500 µg/kg, once)	Increased	
Wu et al., 2011	SD rats, male (6/6)	220–260 g	NS	Borneol, 0.028 g/kg, ig, once	No adjunctive intervention	The brain concentration of HSYA (HSYA, 20.0 mg/kg, ig)	Increased	
Zhang et al., 2011 (2)	Wistar rats, male (3/3)	200 ± 20 g	NS	Borneol, 0.2 g/kg, ig, once daily for 7 d before administration of the drug	Same volume of 50% ethanol, 2.0 ml/kg, ig, once daily for 7 d before administration of the drug	The brain concentration of CBZ (CBZ, 120 mg/kg, ig)	Increased	
Wang et al., 2011	Kunming mice, male and female (9/9)	20 ± 5 g	NS	Borneol, 0.375 g/kg, ig, once	No adjunctive intervention	The brain concentration of jujuboside A (CSJD, 37.5 g/kg, ig)	Increased	
Yu et al., 2011	Kunming mice, male and female (10/10)	18–22 g	NS	Synthetic borneol 0.2, 0.4 g/kg, ig, once daily for 14 d	No ajunctive intervention	1. The brain concentration of EB	Increased	
						2. The brain concentration of Rh 123	Increased	
						3. The permeation index Kp		
						4. The ultrastructure of BBB		
Wu, 2011	Kunming mice, male and female (10/10)	20 ± 2 g	NS	Synthetic borneol, 1 mmol/kg, respectively, ig, once	Same volume of liquid paraffin, ig, once	The brain concentration of EB	Increased	
Dong et al., 2012	SD rats, female (5/5)	180–220 g	10% choral hydrate (3.5 g/kg, ip)	Synthetic borneol, 0.05, 0.1, 0.2, 0.4 g/kg, ig, once at 15 min before administration of the drug	No adjunctive intervention	The brain concentration of geniposide (geniposide, 300 mg/kg, iv)	Increased	
Yu et al., 2012	SD rats, female (5/5)	180–220 g	10% choral hydrate (3.5 g/kg, ip)	Synthetic borneol, 0.2 g/kg, ig, once at 5 min, 15 min or 30 min before administration of the drug	No adjunctive intervention	1. The brain concentration of geniposide (geniposide, 300 mg/kg, iv)	Increased	
						2. The blood concentration of geniposide (geniposide, 300 mg/kg, iv)	ND	
Wang et al., 2012	Kunming mice, NS (6/6)	20 ± 3 g	NS	Synthetic borneol, 0.2 g/kg, ig, once daily for 3 d before administration of the drug	No adjunctive intervention	1. The brain concentration of QUE (QUE, 50 mg/kg, ig)	Increased	
						2. The blood concentration of QUE (QUE, 50 mg/kg, ig)	ND	
Cao, 2013	SD rats, male (5/5)	300 ± 20 g	10% choral hydrate (NS, ip)	Synthetic borneol, 0.125 g/kg ig, once at 30 min before administration of the drug	Same volume of corn embryo oil, ig, once at 30 min before administration of the drug	1. The brain concentration of CPT-11 (CPT-11, 40 mg/kg, CVI)	Increased	
						2. The blood concentration of CPT-11 (CPT-11, 40 mg/kg, CVI)	ND	
Yu et al., 2013	SD rats, male (10/10)	180–220 g	Chloral hydrate (0.3 g/kg, ip)	L-Borneol,0.1, 0.2 g/kg, ig, once daily for 7 d	Same volume of normal saline, ig, once daily for 7 d	1. The brain concentration of Rh 123	Increased	
						2. The permeation index Kp		
						3. The ultrastructure of BBB		
Diao et al., 2013	SD rats, NS (5/5)	250 ± 20 g	10% chloral hydrate (300 mg/kg, ip)	Borneol, 0.7 g/kg, ig, once at 1 h before administration of the drug	Same volume of 75% ethanol, ig, once at 1 h before administration of the drug	The CSF concentration of 131I-MnTBAP (131I-MnTBAP, 1.85MBq per animal, CVI)	Increased	
Huang et al., 2013	Kunming mice, male (8/10)	18–22 g	5% chloral hydrate (NS, NS)	Synthetic borneol, 0.2 g/kg, ig, once daily for 5 d	Same volume of 5% tween and 0.2% CMC-Na, ig, once daily for 5 d	1. The brain concentration of EB	Increased	
						2. The blood concentration of EB	ND	
Zhang, 2014	SD rats, male (6/6)	280 ± 25 g	Chloral hydrate (300 mg/kg, ip)	Synthetic borneol,0.015, 0.030 g/kg, CVI, once	No adjunctive intervention	The brain concentration of kaempferol (kaempferol, 25 mg/kg, CVI)	Increased	
Xin et al., 2014	Wistar rats, male (6/6)	245 ± 10 g	10% choral hydrate (345 mg/kg, ip)	Synthetic borneol,0.186 g/kg, ig, once daily for 7 d before administration of the drug	No adjunctive intervention	1. The brain concentration of Meropenem (Meropenem, 0.208 g/kg, ip)	Increased	
						2. The blood concentration of Meropenem (Meropenem, 0.208 g/kg, ip)	ND	
Liu, 2015	Male and female, New Zealan white (10/10)	2.0–2.5 kg	10% choral hydrate (350–400 mg/kg, iv)	Borneol, 0.002, 0.004, 0.008 g/kg, ie, once	Same volume of solvent, ie, once	The CSF concentration of ligustrazine (ligustrazine, 20 mg per animal, ie)	ND	
Zhang 2015	Male, SD rats (5/5)	255–305 g	NS	Borneol, 15, 30 mg/kg, CVI	No adjunctive intervention	The brain concentration of Kaempferol	Increased	
Guo et al., 2015	Male, SD (5/5)	230–250 g	10% chloral hydrate (3.5 mg/kg, ip) + the mice were injected with 2.5 × 0^6^ C6 cells suspended in 25 ul of PBS (C6/SD glioma model)	Natural Borneol, 140, 35 mg/kg, ig, once at 1 h before administration of the drug	Same volume of CMC ig, once at 1 h before administration of the drug	Effect of bonenol on pharmacokinetic parameters of methotrexate in brain	Increased	
Yu et al., 2015	Male, NIH rats (10/10)	26–30 g	NS	Borneol, 50, 100, 200 mg/kg, ig, twice daily for 7 d	No adjunctive intervention	1. The brain concentration of Rh 123 (Rh 123, 0.3 mg/kg, CVI )	Increased	
						2. The blood concentration of Rh 123 (Rh 123, 0.3 mg/kg, CVI )	ND	
Zhao et al., 2015	Female and male, Wistar rats (8/8)	18–22 g	NS	Borneol, 3 mg/kg, ig, once daily for 7 d	No adjunctive intervention	The brain concentration of nerve growth factor	Increased	
Ren, 2016	Male, Wistar rats (13/13)	200 ± 20 g	NS	Natural Borneol, 14.28 g/kg, ig, once daily for 10 d	No adjunctive intervention	The brain concentration of phenytoin sodium	Increased	
Wei, 2016	Kunming mice, female (5/5)	20–25 g	Ether (NS, NS)	Natural Borneol, 125, 250 mg/kg, ig, 30 min before administration of the drug	Same volume of 75% alcohol 0.01 ml/g, ig, 30 min before administration of the drug	1. The brain concentration of Erlotinib (Erlotinib, 50 mg/kg, ig)	Increased	
Wu, 2016	FVB rats, NS (6/6)	NS	1.25% avertin (NS, NS)	Natural Borneol, 2 mg/10g, ig, once at 1 h before measuring	No adjunctive intervention	1. The brain concentration of Rh 123	Increased	
						2. The brain concentration of Adriamycin (Adriamycin, 58 ug/10 g, iv)	Increased	
Tang et al., 2016	Kunming mice, female and male (54/54)	NS	NS + co-culture of primary brain microvessel endothelial cells and astrocytes in rats	Natural Borneol, 25, 50, 100 mg/kg, ig, once before administration of the drug	Same volume of 50% alcohol, once before administration of the drug	The brain concentration of puerarin	Increased	
Hou et al., 2016	SD rats, male (36/36)	180–220 g	Chloral hydrate (NS, ip)	Borneol, 100 mg/kg, ig and iv	No adjunctive intervention	The brain concentration of asiaticoside	Increased	
Yin, 2017	C57BL/6 mice, male (10/10)	20 ± 2 g	NS + the mice were injected with 5 × 10^4^ GL261 cells suspended in 4ul of PBS (mouse GL261 glioma models)	L-Borneol, 0.1, 0.15, 0.3, 0.6, 0.9 g/kg, ig, once at 1 h before administration of the drug	No adjunctive intervention	1. The brain concentration of Cisplatin	Increased	
						2. The brain concentration of EB	Increased	
						3. Survival of tumor-bearing mice		
						4. Gadolinium-enhancement ratio		

BBB: the blood–brain barrier; increased: an significantly increasing blood–brain barrier permeability after the administration of borneol; decreased: an significantly decreasing blood–brain barrier permeability after the administration of borneol; ND: no statistical difference between treatment and control group; Increased? or decreased?, the efficacy result was reported as increasing or decreasing blood brain barrier permeability with absence of statistical analysis or available original data; NS: not stated; AI: acupoint injection; EB: Evans blue; Sul: sulfanilamide; ig: intragastric administration; ip: intraperitoneal administration; in: intranasal administration; iv: intravenous injection; Rh 123: rhodamine 123; *Kp*: the permeation index calculated by the ratio of Rh 123brain/Rh 123blood; VCR: Vincristine; CVI: caudal vein injection; P-gp, P-glycoprotein; TMP: tetramethylpyrazine; CSF: cerebrospinal fluid; NT-NP: neurotoxin nanoparticle; CSJD: Compound Shuyu Jiannao Decoction; TMPP: tetramethylpyrazine phosphate; SF: sodium ferulate; SV: Sodium Valproate; CBZ: carbamazepine; Rif: rifampicin; AZTP-CL: azidothymidine palmitate liposome; AZT: azidothymidine; CPT-11: Irinotecan; HSYA: hydroxysafflor yellow A; ACNU: nimustine; TMZ: Temozolomide; QUE: quercetin; CT: computed tomography; Vs: Valproate sodium; Hup: Huperzine, CMC: carboxymethylcellulose sodium.

### 3.3. Quality of included study

The quality scores of studies included varied from 1 to 5 out of 10 points with the average of 2.8. Among them, 1 study scored 1 point; 22 studies scored 2 points; 24 studies scored 3 points; 8 studies scored 4 points; 3 studies scored 5 points (Table 2). Forty-seven studies were peer-reviewed publication and 11 studies were Master’s thesis or PhD thesis. Six studies described the control of temperature. Forty-seven studies declared the random allocation. Forty-five studies described the use of anesthetic without significant intrinsic neuroprotective activity. Sixteen studies stated the compliance with animal welfare regulations. Three studies described the application of animal or model with relevant comorbidities. None of the studies included reported the masked conduct of experiments, the blinded assessments of outcome, a sample size calculation or a statement of potential conflict of interests.

**Table 2.** Quality assessment of included studies.

Study	A	B	C	D	E	F	G	H	I	J	Total
Wang et al., 1992	+	–	–	–	–	+	–	–	–	–	2
Liang et al., 1993	+	–	–	–	–	+	–	–	–	–	2
Liu et al., 1994	+	–	+	–	–	+	–	–	–	–	3
Xu & Wang 1995	+	–	+	–	–	+	–	–	–	–	3
Dong et al., 2002	+	–	+	–	–	+	–	–	–	–	3
Lin et al., 2003	+	–	–	–	–	+	–	–	–	–	2
Jia et al., 2004	+	–	–	–	–	+	–	–	–	–	2
Wu et al., 2004	+	–	+	–	–	+	–	–	–	–	3
Zhang et al., 2005	+	–	+	–	–	+	–	–	–	–	3
Chen 2005	–	–	+	–	–	+	–	–	–	–	2
Zhou et al., 2005	+	–	+	–	–	+	–	–	–	–	3
Wang 2006	–	–	–	–	–	+	–	–	+	–	2
Wang et al., 2006	+	–	+	–	–	+	–	–	–	–	3
Zheng et al., 2007	+	–	+	–	–	+	–	–	–	–	3
Xiao et al., 2007	+	–	+	–	–	+	–	–	+	–	4
Chen et al., 2007	+	–	+	–	–	+	–	–	–	–	3
Zhang et al., 2007	+	–	+	–	–	+	–	–	–	–	3
Liu & Gao [40]	+	–	+	–	–	+	–	–	–	–	3
Lin et al., 2008	+	–	+	–	–	–	–	–	–	–	2
Liu et al., 2008	+	–	+	–	–	–	–	–	–	–	2
Zhou et al., 2008	+	–	+	–	–	–	–	–	–	–	2
Shi & Zhao 2008	+	–	+	–	–	+	–	–	–	–	3
Li et al., 2008	–	–	+	–	–	+	–	–	–	–	2
Liu et al., 2008	+	–	+	–	–	–	–	–	–	–	2
Ge et al., 2008	+	–	–	–	–	+	–	–	–	–	2
Gao et al., 2009	+	–	+	–	–	+	–	–	–	–	3
Wu et al., 2009	+	–	–	–	–	+	–	–	–	–	2
Wang et al., 2009	+	–	+	–	–	+	–	–	–	–	3
Xiao & Ping 2009	+	–	–	–	–	+	–	–	–	–	2
Chai et al., 2009	+	+	+	–	–	+	–	–	+	–	5
Zhu 2009	–	–	+	–	–	+	–	–	+	–	3
Wei et al., 2010	+	–	+	–	–	+	–	–	+	–	4
Zhang et al., 2011 (1)		–	+	–	–	+	–	–	+	–	3
Wu 2011	+	–	+	–	–	–	–	–	+	–	3
Zhang et al., 2011 (2)	+	–	+	–	–	+	–	–	–	–	3
Wang et al., 2011	+	–	+	–	–	–	–	–	–	–	2
Yu et al., 2011	+	–	–	–	–	+	–	–	–	–	2
Wu et al., 2011	+	–	+	–	–	–	–	–	–	–	2
Dong et al., 2012	+	–	+	–	–	+	–	–	+	–	4
Yu et al., 2012	+	–	+	–	–	+	–	–	+	–	4
Wang et al., 2012	+	–	+	–	–	–	–	–	+	–	3
Cao 2013	–	–	+	–	–	+	–	–	+	–	3
Yu et al., 2013	+	–	+	–	–	+	–	–	+	–	4
Diao et al., 2013	+	–	+	–	–	+	–	–	–	–	3
Huang et al., 2013	+	–	+	–	–	+	–	–	–	–	3
Zhang, 2014	+	+	+	–	–	+	–	–	+	–	5
Xin et al., 2014	+	–	+	–	–	+	–	–	+	–	4
Liu 2015	–	–	–	–	–	+	–	–	–	–	1
Zhang 2015	+	+	+	–	–	–	–	–	+	–	4
Guo et al., 2015	+	–	+	–	–	+	+	–	–	–	4
Yu et al., 2015	+	–	+	–	–	–	–	–	–	–	2
Zhao et al., 2015	+	+	–	–	–	–	–	–	–	–	2
Ren 2016	–	+	+	–	–	–	–	–	–	–	2
Wei 2016	–	–	+	–	–	+	–	–	–	–	2
Wu 2016	–	–	+	–	–	+	–	–	–	–	2
Tang et al., 2016	+	–	+	–	–	–	+	–	–	–	3
Hou et al., 2016	+	–	+	–	–	+	–	–	–	–	3
Yin 2017	+	+	+	–	–	–	+	–	+	–	5

A: peer-reviewed publication; B: monitoring of physiological parameters such as temperature; C: random allocation; D: blinded conduct of the experiments; E: blinded assessment of outcome; F: use of anesthetic without significant intrinsic neuroprotective activity (e.g. ketamine); G: animal and/or model (brain tumor model, epilepsy, intracranial infection, cognitive dysfunction or Parkinson); H: sample size calculation; I: compliance with animal welfare regulations; J: statement of potential conflict of interests.

### 3.4. Effectiveness

#### 3.4.1. Co-administration of drug concentrations in CNS

Forty-seven studies reporting the assessments of co-administration of drug concentrations in CNS, of which 45 studies showed the significant effects of borneol for improving CNS drug delivery and 2 studies showed no difference (Chen, 2005; Liu, 2015). Among the 45 studies, several main categories of drugs were reported, including antineoplastic drugs, antibiotics, antiviral drugs, drugs for epileptic, Parkinsonism and cognition. Some Chinese herbal medicines also were mentioned. Eight types of the drugs were reported more than once. There studies investigated the effect of borneol on tetramethylpyrazine concentration-curve in brain tissue (Wang et al., 2006; Li et al., 2008; Xiao & Ping, 2009) and in CSF (Liu, 2015); three studies (Dong et al., 2002; Jia et al., 2004; Yin et al., 2017) on the brain concentration of cisplatin; two studies on the brain concentration (Gao et al., 2009) and on the CSF concentration of methotrexate (Guo et al., 2015); two studies (Zhang, 2014; Zhang et al., 2015) on the brain concentration of Kaempferol; two studies (Zhou et al., 2008; Zhang et al., 2011) on the brain concentration and one study (Zhou et al., 2005) on the CSF to serum concentration ratio of carbamazepine over time; three studies (Chen et al., 2007; Zhang et al., 2007; Liu & Gao, 2007) on CSF concentration-curve of valproate; two studies (Dong et al., 2012; Yu et al., 2012) on the main pharmacokinetic parameters of geniposide in brain tissue; two studies on the brain concentration (Liu et al., 2008) and the CSF to serum concentration ratio (Zheng et al., 2007) of *Salvia miltiorrhiza* over time (Table 3).

**Table 3.** The classification of drugs transferred into the brain.

Antineoplastic drugs	Antibiotics and Antiviral drugs	Drugs for epileptic, Parkinson, and cognition	Traditional Chinese medicine	Other drugs
Cisplatin	Gentamicin	Carbamazepine	Ligustrazine	^131^I-MnTBAP
Dong et al., 2002	Liu et al., 1994	Zhou et al., 2005	Wang et al., 2006	Diao et al., 2013
Jia et al., 2004	Sulfanilamide	Zhou et al., 2008	Li et al., 2008	Nerve growth factor
Yin 2017	Xu & Wang 1995	Zhang et al., 2011 (2)	Xiao & Ping 2009	Zhao et al., 2015
Nimustine	Rifampicin	Sodium Valproate	Liu 2015	Fentanyl
Shi & Zhao 2008	Wu et al., 2004	Chen et al., 2007	Salvia miltiorrhiza	Wang 2006
Methotrexate	Clindamycin	Zhang et al., 2007	Zhang et al., 2007	As_2_O_3_
Gao 2009	Wang 2006	Liu & Gao 2007	Liu et al., 2008	Xiao et al., 2007
Guo et al., 2015	Cefatriaxone	Phenytoin sodium	Ginsenoside	Neurotoxin nanoparticle
Vincristine	Wei et al., 2010	Ren 2016	Wang et al., 2009	Chai et al., 2009
Zhu 2009	Meropenem	Amantadine Hydrochloride	Ferulic acid	
Irinotecan	Xin et al., 2014	Wang 2006	Lin et al., 2008	
Cao 2013	Azidothymidine	Huperzine	Puerarin	
Erlotinib	Wu et al., 2009	Zhang et al., 2011 (1)	Tang et al., 2016	
Wei 2016			Asiaticoside	
Adriamycin			Hou et al., 2016	
Wu 2016			HSYA	
Quercetin			Wu et al., 2011	
Wang et al., 2012			Jujuboside	
Kaempferol			Wang et al., 2011	
Zhang 2014			Geniposide	
Zhang et al., 2015			Dong et al., 2012	
			Yu et al., 2012	
			Paeonol	
			Chen 2005	

HSYA: hydroxysafflor yellow A; ^131^I-MnTBAP: manganese porphyrin labeled by ^131^I.

#### 3.4.2. BBB permeability and meta-analysis

**Figure 2.**
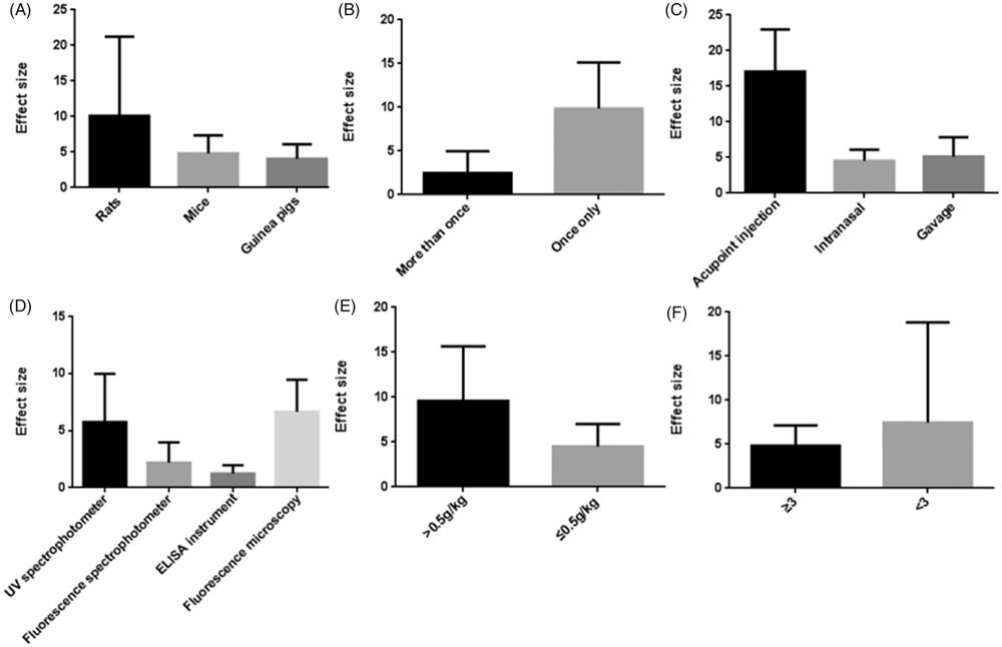
Subgroup analysis according to brain Evans blue content. (A) animal species; (B) the frequency of borneol administration; (C) the mode of application; (D) the instrument used for quantification; (E) the therapeutic dose of borneol; (F) the quality of studies. The vertical axis represents effect size point estimates for borneol and 95% confidence intervals.

**Figure 3.**
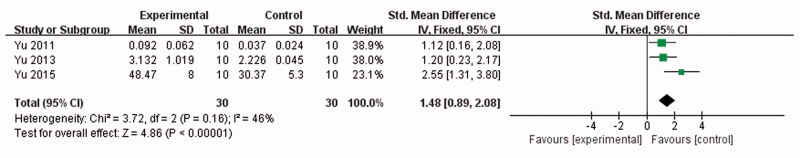
The forest plot: effects of borneol for improving the BBB permeability compared with control group according to brain Rh 123 content.

Nine studies (Liang et al., 1993; Xu & Wang, 1995; Lin et al., 2003; Zhang et al., 2005; Zhu, 2009; Yu et al., 2011; Wu et al., 2011; Huang et al., 2013; Yin et al., 2017) used EB content as outcome measures to test the BBB permeability and involved following 11 comparisons: 8 comparisons (Xu & Wang, 1995; Lin et al., 2003; Zhang et al., 2005; Zhu, 2009; Yu et al., 2011; Wu et al., 2011; Huang et al., 2013; Yin et al., 2017) with increased effects (*p*<.05), 1 comparison (Xu & Wang, 1995) with no difference (*p*>.05), and 2 comparisons (Liang et al., 1993) listed as “increased?” without data. Meta-analysis of 8 (26,28,31,53,59,60,67,80) comparisons with available data showed significant effects of borneol for increasing brain EB content compared with control (*n* = 141, SMD 5.85, 95% CI: 3.56 ∼ 8.14, *p*<.00001). There was high heterogeneity among these 8 comparisons (*χ*^2^ = 87.54, *p*<.00001, *I*^2^ = 92%). Thus, subgroup analysis was followed according to stratification on animal species, the frequency of administration, the mode of application, the dose of administration and the instrument used for quantification of brain EB content. In the subgroup analyses of these factors, the effect size of rat species was larger than other two animal mice and guinea pigs species (SMD = 11.59 vs. SMD = 4.27 vs. SMD = 4.79, Figure 2(A)). The effect size of single administration animals was greater than successive administration animals (SMD = 9.11 vs. SMD = 2.72, Figure 2(B)). The mode of application showed great discrepancy in the overall effect of outcome measure, which the administration by acupoint injection with only scale of 7.2% weight accounted for greater effect size than by intranasal administration and gavage (SMD = 17.55 vs. SMD = 4.79 vs. SMD = 4.77, Figure 2(C)). The effect size was greater in animals using fluorescence microscopy than in animals using other quantified method, including UV spectrophotometer, fluorescence spectrophotometer, ELISA instrument (Figure 2(D)). The group that the therapeutic dose of borneol larger than 0.5 g/kg showed greater effect size than the group with 0.5 g/kg or less dose (SMD = 9.37 vs. SMD = 3.93, Figure 2(E)). The lower quality studies exhibit larger effect size than the higher ones (SMD = 9.38 vs. SMD = 4.68, Figure 2(F)). Four studies (Yu et al., 2011; Yu et al., 2013; Yu et al., 2015; Wu, 2016) used Rh 123 content as outcome measures to test the BBB permeability, after removing 1 study (Wu, 2016) for concentration-curve of Rh 123, meta-analysis of three studies (Yu et al., 2011, 2013, 2015) indicated that borneol can improve Rh123 concentration in CNS significantly compared with control (*n* = 30, SMD 1.48, 95% CI: 0.89 ∼ 2.08, *p*<.00001). There was low heterogeneity among the three included studies (*χ*^2^ = 3.72, *p* = .16, *I*^2^ = 46%) (Figure 3). Compared with controls, two studies (Wang et al., 1992; Zhang, 2011) showed significant effects of borneol for increasing brain imaging agent entering the brain (*p*<.05) but failed to obtain primary data for poor analysis, one study (Wang et al., 2011) for increasing brain water content (*p*<.05), four studies (Zhang et al., 2007; Ge et al., 2008; Yu et al., 2011, 2013) for increasing the opening effects of the ultrastructure of BBB (*p*<.05).

#### 3.4.3. The safety of co-administration of borneol

Six studies (Wu et al., 2009; Yu et al., 2012; Cao, 2013; Diao et al., 2013; Xin et al., 2014) indicated that the increased effects of borneol on brain or CSF drug concentration were accompanied by the absence of an increase in the blood drug concentration. One study (Ge et al., 2008) reported that the opening of BBB by borneol has been found to be reversible and physiological in accordance with the ultrastructure assessments of BBB, which could last up to 8 h after its intragastric administration in rats.

#### 3.4.4. Possible mechanisms

The possible mechanisms of borneol in an increase of BBB permeability are summarized as follows: (Abbott, 2013) inhibition of drug efflux through combining with P-gp competitively and inhibiting its activity (Xiao et al., 2007; Chen et al., 2007; Zhu, 2009; Yu et al., 2011; Wang et al., 2012; Diao et al., 2013; Yu et al., 2015; Ren, 2016; Tang et al., 2016; Yin et al., 2017) and decreasing the expressions of both Mdr1a, Mdr1b, and Mrp1 in hippocampus and hypothalamus (Yu et al., 2013); (Zlokovic, 2008) increasing the amount of 5-hydroxytryptamine and histamine (Xiao et al., 2007; Chen et al., 2007; Zhang et al., 2011; Wang et al., 2012; Cao, 2013; Diao et al., 2013; Yin et al., 2017) in the hypothalamus; (Abbott et al., 2010) improvement of the circulation by enhancing the expression of NO *via* up-regulating the expression of NOS (Chen, 2005; Xiao et al., 2007; Chen et al., 2007; Zhou et al., 2008; Yu et al., 2011; Diao et al., 2013); (Pardridge, 2005) releasing tight junction between capillary endothelial cells (Wang et al., 2009; Chai et al., 2009; Yu et al., 2011); (Demeule et al., 2002) inhibiting the permeability of a chloride-permeable channel CIC-3 (77) (Figure S1).

## 4. Discussion

### 4.1. Summary of evidence

This is the first preclinical systematic review to determine the effects of borneol on CNS drug delivery in animal models. Fifty-eight with 1137 animals were selected. The quality of studies included was generally medium. The evidence available from this study showed that the co-administration of borneol is a promising candidate for CNS drug delivery. The effects of borneol are closely associated with the inhibition of efflux protein function, releasement of tight junction protein, increasement of vasodilatory neurotransmitters, and inhibition of active transport by ion channels.

### 4.2. Limitations

Our study only included two animal species, rodent, and rabbit, which may potentially impose restrictions on the promotion of the findings. The significant heterogeneity across studies indicates that conclusions should have been treated more cautious. The methodological quality of studies included was generally moderate, which is an inherent drawback in the primary study. It was indicated that a lack of blinding outcome assessments attributed to a 27% overestimation of the mean reported effect size (Holman et al., 2015). No study reported the data on a sample size calculation, which may inflate the reported effect size. Therefore, the results in this study should be interpreted with caution.

### 4.3. Implications

In this study, the findings showed the enhanced penetration of a variety of drugs acting on the CNS and increased BBB permeability of EB and Rh 123 after the co-administration of borneol. Thus, we proposed accordingly the co-administration of borneol as a potential approach for effective brain drug delivery with several advantages. First, the administration of borneol is noninvasive and allows for repeated applications by gavage, intravenous injection, and nasal administration. Second, the increased effects of borneol on brain or CSF drug concentration were accompanied by the absence of an increase in the blood drug concentration (Wu et al., 2009; Yu et al., 2012; Cao, 2013; Diao et al., 2013; Xin et al., 2014), which indicated that the co-administration of borneol did not increase the risk of peripheral adverse effects. Third, the opening of BBB by borneol has been found to be reversible and physiological in accordance with the ultrastructure assessments of BBB, which could last up to 8 h after its intragastric administration in rats (Ge et al., 2008) and did not cause an up-regulation of inducible nitric oxide synthase (Baoshe & Qi de, 2002), the over-expression that always occurred in the presence of pathological processes, e.g. Hypoxia (Robinson et al., 2011). Thus, the co-administration of borneol may be a safe and promising strategy for effective BBB penetration enhancer for CNS drug.

The evidence of mechanisms available from this study showed that borneol enhanced BBB permeability largely through inhibiting efflux protein function, releasing tight junction protein, increasing vasodilatory neurotransmitters, inhibiting active transport by ion channels. Moreover, some studies (Zhang et al., 2012; Li et al., 2012) reported that borneol can increase the levels of excitatory amino acid greater than the levels of inhibitory amino acids increased in the whole brain, leading to a transient elevation in the excitation ratio, which was conjectured as a reason of the transient and reversible effects of borneol on enhancing BBB permeability. Thus, borneol for opening BBB permeability transiently and reversibly depended on multi-targeted mechanisms.

## 5. Conclusions

Our findings indicate that borneol is a multi-targeted BBB permeability mediator, suggesting that the co-administration of borneol is a promising candidate for CNS drug delivery. The effects of borneol are closely associated with the inhibition of efflux protein function, the releasement of the tight junction protein, increasement of vasodilatory neurotransmitters, and inhibition of active transport by ion channels.

## Supplementary Material

Supplemental_File.pdf
