# Supplementary material for: Borneol, a messenger agent, improves central nervous system drug delivery through enhancing blood–brain barrier permeability: a preclinical systematic review and meta-analysis
Source: Drug Deliv. 2018 Oct 18;25(1):1617–33. doi: 10.1080/10717544.2018.1486471 (PMC6225363; doi:10.1080/10717544.2018.1486471)
Supplement: Supplemental_File.pdf [file IDRD_A_1486471_SM6901.pdf]

Supplemental File

Figure S1

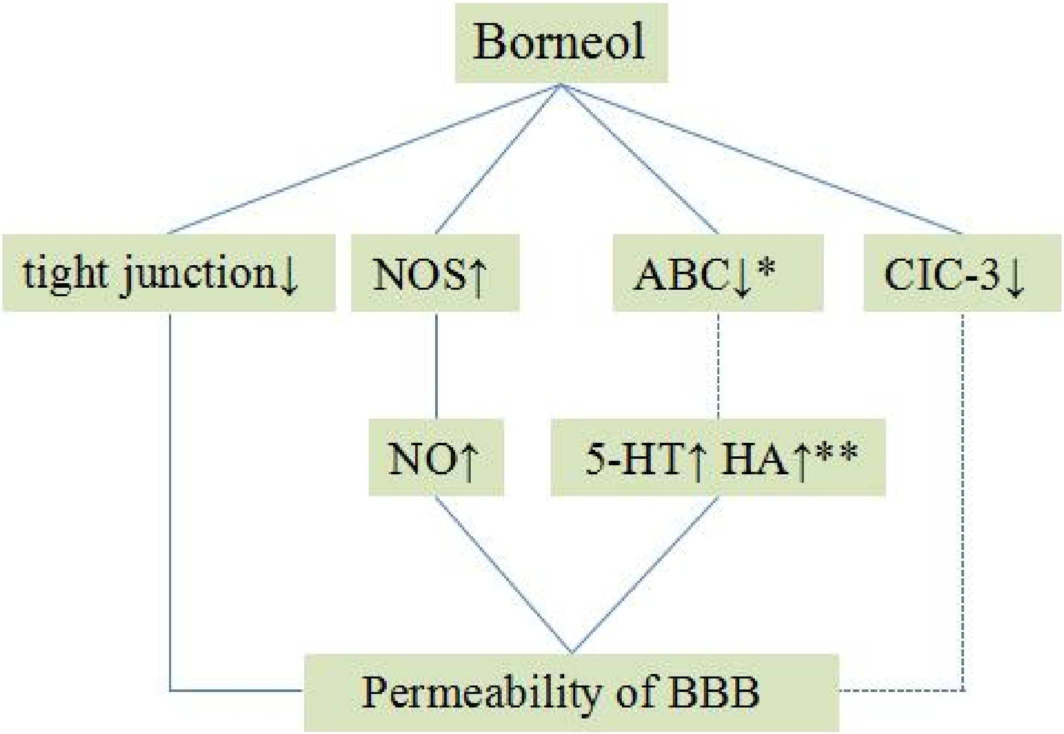

Figure S1: A schematic representation of possible mechanisms of borneol for improving the BBB permeability. Solid lines indicate established effects, whereas dashed lines represent putative mechanisms. *ABC, ATP-binding cassette, including P-gp, Mdr1a, Mdr1b and Mrp1; **5-hydroxytryptamine and histamine in the hypothalamus; BBB; blood-brain barrier.
